# Comment on “Manifestations of Sasang Typology according to Common Chronic Diseases in Koreans”

**DOI:** 10.1155/2020/8706183

**Published:** 2020-06-19

**Authors:** Han Chae

**Affiliations:** School of Korean Medicine, Pusan National University, 49 Busandaehak-ro, Mulgeum-eup, Yangsan-si, Gyeongsangnam-do 50612, Republic of Korea

This is a comment on “Manifestations of Sasang Typology according to Common Chronic Diseases in Koreans” recently published by Hong et al. [1] that examined the psychological, physical, and clinical features of chronic diseases such as diabetes (DM), hypertension, functional dyspepsia (FD), major depressive disorder (MDD), and adenomyosis in university hospital outpatients using Sasang Personality Questionnaire (SPQ) [2], Sasang Digestive Function Inventory (SDFI) [3], and BMI. The diagnosis and recruitment of various chronic disease outpatients are well organized, but the lack of review on SPQ studies and a miscalculation of SDFI limited the results and discussion.

As for the SPQ measuring biopsychological characteristics of Yin-Yang personality traits, the SPQ and its subscale scores of DM, hypertension, FD, and MDD were correctly illustrated, but their implications were not discussed in the context of recently published reports [4, 5]. The SPQ-B score of the MDD group was significantly lower than DM, hypertension, FD, and adenomyosis groups [1]. Considering that a person with a low SPQ-B score has an introverted, inactive, asocial, unenergetic, and inhibited personality with low vitality and low positive affection [4, 5], the SPQ-B score would be a good psychological measure for differential diagnosis of MDD patients in traditional Korean medicine.

The SPQ-E score of the FD group was significantly higher than that of the hypertension and adenomyosis groups, and the MDD group was similar to the FD group [1]. A person with high SPQ-E score has an unstable, irritable, intolerant, pessimistic, and emotional personality which might make him/her vulnerable to the psychopathological issues of MDD and FD [4, 5]. Since the SPQ-B and SPQ-E have contradictory clinical effects, there was no significant difference in SPQ-Total score between chronic disease groups of Hong's study [1] that confirms the conclusions of previous studies [4, 5].

As for the SDFI measuring good digestive function and appetite, the article of Hong et al. [1] made critical mistakes. The SDFI-D was reversely calculated as representing problematic poor digestive function, and the SDFI-A and SDFI-E were correctly calculated with erroneous operational definitions. Subsequently, the SDFI-Total as a sum of SDFI-D, SDFI-A, and SDFI-E was miscalculated, and this affects the clinical usefulness of SDFI and its subscales in Sasang typology [6].

The operational definition of SDFI-D is a measure of good and hyperactivated digestive function, and a person with high SDFI-D should have a high BMI [3, 6–8]. A previous clinical study showed that the SDFI-D has a negative correlation with Nepean Dyspepsia Index-Korean (*r* = −0.585) and Functional Dyspepsia-Related Quality of Life (*r* = −0.433), while the SDFI-E (tendency of high eating speed and large meal volume) has a positive correlation with Dutch Eating Behavior Questionnaire (*r* = 0.481) [3]. As for the anthropometric body shape, the BMI has a significantly positive correlation with SDFI-Total (*r* = 0.299) and SDFI-D (*r* = 0.310) scores [3].

In the article by Hong et al. [1], the BMI of DM (24.91 ± 3.77) and hypertension (25.44 ± 3.07) groups was reported to be higher than that of FD (22.81 ± 2.73), MDD (23.37 ± 3.88), and adenomyosis (22.54 ± 3.55) groups; however, the SDFI-D of DM (10.76 ± 8.73) and hypertension (10.81 ± 6.80) groups was lower than that of FD (18.89 ± 9.62), MDD (16.56 ± 9.34), and adenomyosis (16.58 ± 7.99) groups. These SDFI-D scores of Hong's study seems to be reversely calculated, and the presumed and correct SDFI-D scores of DM (29.24 ± 8.73) and hypertension (29.19 ± 6.80) groups would be higher than that of FD (21.11 ± 9.62), MDD (23.44 ± 9.34), and adenomyosis (23.42 ± 7.99) groups which would be consistent with previous clinical reports [3, 8].

From this perspective, the high score of SDFI-D would be a typical clinical feature of metabolic disease showing DM, hypertension, and obesity (high BMI) distinguished from other chronic diseases. The FD patients with low SDFI-D and high SPQ-E scores might be recognized as bad or hypoactive digestive function along with psychopathological vulnerability in traditional Korean medicine.

The SDFI-A score of MDD group was significantly lower than that of DM, hypertension, FD, and adenomyosis groups in Hong's study [1]. Considering the results that the MDD group has low SPQ-B and high SPQ-E, the MDD patients might be identified with low SDFI-A (decreased appetite, feeling of hunger, and pleasure of eating), low SPQ-B (inactive and asocial personality with low vitality), and high SPQ-E (unstable, intolerant, and emotional personality with psychopathological vulnerable) in traditional medicine clinics dealing with chronic diseases [2, 3].

As a conclusion, with consideration of the latest clinical studies [4–7], the clinical usefulness of SPQ and SDFI subscales in chronic diseases should be acknowledged as that the MDD patients have high SPQ-E, low SPQ-B, and low SDFI-A; FD patients have high SPQ-E and low SDFI-D; and metabolic disease patients of hypertension, DM, and obesity have high SDFI-D which were missed out in Hong's article [1].
